# Constructing Lipoparticles Capable of Endothelial Cell-Derived Exosome-Mediated Delivery of Anti-miR-33a-5p to Cultured Macrophages

**DOI:** 10.3390/cimb45070355

**Published:** 2023-07-04

**Authors:** Jing Echesabal-Chen, Kun Huang, Lucia Vojtech, Olanrewaju Oladosu, Ikechukwu Esobi, Rakesh Sachdeva, Naren Vyavahare, Hanjoong Jo, Alexis Stamatikos

**Affiliations:** 1 Department of Food, Nutrition and Packaging Sciences, Clemson University, Clemson, SC 29634, USA; jchen11@clemson.edu (J.E.-C.); kunh@g.clemson.edu (K.H.); oolados@g.clemson.edu (O.O.); iesobi@g.clemson.edu (I.E.); 2 Department of Obstetrics & Gynecology, University of Washington, Seattle, WA 98109, USA; luciav@uw.edu; 3 Department of Chemistry, Clemson University, Clemson, SC 29634, USA; rakeshs@clemson.edu; 4 Department of Bioengineering, Clemson University, Clemson, SC 29634, USA; narenv@clemson.edu; 5 Coulter Department of Biomedical Engineering, Georgia Institute of Technology and Emory University, Atlanta, GA 30322, USA; hjo@emory.edu

**Keywords:** antagomiR, HDL, intima, microRNA, nanoparticle, reverse cholesterol transport, vascular inflammation

## Abstract

Atherosclerosis is driven by intimal arterial macrophages accumulating cholesterol. Atherosclerosis also predominantly occurs in areas consisting of proinflammatory arterial endothelial cells. At time of writing, there are no available clinical treatments that precisely remove excess cholesterol from lipid-laden intimal arterial macrophages. Delivery of anti-miR-33a-5p to macrophages has been shown to increase apoAI-mediated cholesterol efflux via ABCA1 upregulation but delivering transgenes to intimal arterial macrophages is challenging due to endothelial cell barrier integrity. In this study, we aimed to test whether lipoparticles targeting proinflammatory endothelial cells can participate in endothelial cell-derived exosome exploitation to facilitate exosome-mediated transgene delivery to macrophages. We constructed lipoparticles that precisely target the proinflammatory endothelium and contain a plasmid that expresses XMOTIF-tagged anti-miR-33a-5p (LP-pXMoAntimiR33a5p), as XMOTIF-tagged small RNA demonstrates the capacity to be selectively shuttled into exosomes. The cultured cells used in our study were immortalized mouse aortic endothelial cells (iMAECs) and RAW 264.7 macrophages. From our results, we observed a significant decrease in miR-33a-5p expression in macrophages treated with exosomes released basolaterally by LPS-challenged iMAECs incubated with LP-pXMoAntimiR33a5p when compared to control macrophages. This decrease in miR-33a-5p expression in the treated macrophages caused ABCA1 upregulation as determined by a significant increase in ABCA1 protein expression in the treated macrophages when compared to the macrophage control group. The increase in ABCA1 protein also simulated ABCA1-dependent cholesterol efflux in treated macrophages—as we observed a significant increase in apoAI-mediated cholesterol efflux—when compared to the control group of macrophages. Based on these findings, strategies that involve combining proinflammatory-targeting lipoparticles and exploitation of endothelial cell-derived exosomes appear to be promising approaches for delivering atheroprotective transgenes to lipid-laden arterial intimal macrophages.

## 1. Introduction

Atherosclerosis is a chronic condition that causes narrowing of the arteries due to plaque formation [1,2]. The most deleterious consequence of atherosclerosis is a vulnerable plaque rupture causing a thrombus to form that can occlude the arterial lumen and possibly lead to death from a myocardial infarction or ischemic stroke [3,4]. The two major drivers of atherosclerosis are arterial cholesterol accumulation and vascular inflammation [5,6]. For the latter, it is critical to emphasize that vascular inflammation in the context of atherosclerosis does not occur systemically, but in atheroprone areas [7,8]. Indeed, atherosclerotic inflammation is stimulated by endothelial dysfunction and induced by endothelial activation [9], which can cause both atherogenesis and atherosclerosis progression [10,11,12].

One of the most crucial proinflammatory adhesion molecules expressed by the endothelium when endothelial activation occurs is VCAM-1 [13]. A main function of VCAM-1 is transendothelial migration of circulatory monocytes into the intima [14,15], whereby monocytes differentiate into macrophages and engulf LDL that has entered the intima, which can cause atherosclerosis development [16,17,18]. Therefore, VCAM-1 expression within endothelial cells is thought to trigger atherosclerosis and it is well recognized that robust endothelial VCAM-1 expression predominantly occurs in atheroprone regions and atherosclerotic areas, but not in atheroresistant endothelial arterial cells [19]. 

Exploiting endothelial VCAM-1 expression as a target for atheroprotective therapy has previously been successful [20]. Indeed, decorating nanoparticles with the VCAM-1-binding peptide VHPK results in nanoparticles being precisely internalized by inflamed endothelial cells [21,22]. Furthermore, data have shown that atherosclerosis can be prevented in atherogenic mice that are administered VHPK-decorated nanoparticles containing antiatherogenic anti-miR-712 [21]. However, this atheroprotective effect observed from VHPK-decorated, nanoparticle-based anti-miR-712 delivery appears to be confined to inflamed endothelia [21]. Thus, a possible way to maximize the atheroprotective benefit of nanoparticles is for effective delivery of atheroprotective transgenes to lipid-laden intimal macrophages, which is a major cell type responsible for the development of atherosclerotic plaque formation [23].

Gene delivery to intimal and other subendothelial cells is extremely challenging due to endothelial cells acting as a barrier that impedes transgenic subendothelial entry [24,25]. Therefore, nanoparticles and viral vectors are unlikely to cross the endothelium and enter the intima to deliver atheroprotective transgenes to lipid-laden intimal macrophages. A possible strategy to overcome this limitation of atheroprotective gene-based therapy is utilizing endothelial cell-derived exosomes to deliver transgenes to intimal cells. Indeed, incorporating an XMOTIF sequence to small RNA results in small RNA species being selectively packaged into exosomes [26,27,28].

We previously used this XMOTIF technology to generate a helper-dependent adenoviral vector (HDAd) that expresses XMOTIF-tagged anti-miR-33a-5p (HDAdXMoAntimiR33a5p) [28]. Since miR-33a-5p is considered proatherogenic via silencing ABCA1 expression resulting in impaired apoAI-mediated cholesterol efflux, inhibiting miR-33a-5p is considered atheroprotective via augmenting intracellular cholesterol removal, with data supporting this notion [29,30,31]. When cultured endothelial cells are transduced with HDAdXMoAntimiR33a5p, this results in enhanced exosomal uploading of anti-miR-33a-5p into exosomes released by transduced endothelial cells. Moreover, when these anti-miR-33a-5p-filled exosomes are exposed to cultured macrophages, this results in an increase in both ABCA1 protein expression and apoAI-mediated cholesterol efflux in treated macrophages [28].

Based on the prior mentioned successes of nanoparticle- and exosome-based atheroprotective therapies, we attempted to combine these two approaches to possibly allow inflamed endothelial cells to package anti-miR-33a-5p into exosomes that have the potential to be internalized by lipid-laden intimal macrophages. Thus, in this study, we aimed to test the hypothesis that inflamed cultured endothelial cells exposed to VCAM-1-targeting lipoparticles (LPs) containing a plasmid that expresses XMOTIF-tagged anti-miR-33a-5p (LP-pXMoAntimiR33a5p) increases ABCA1-dependent cholesterol efflux in cultured macrophages via endothelial cell-derived exosome-mediated transfer of anti-miR-33a-5p.

## 2. Materials and Methods

### 2.1. Tissue Culture

Immortalized mouse aortic endothelial cells (iMAECs) [32] were cultured and maintained in conditions previously described [33]. The RAW 264.7 macrophage cell line was purchased from ATCC (Manassas, VA, USA) and grown in medium that contained the following: high-glucose Dulbecco’s modified Eagle’s medium (DMEM; Corning, New York, NY, USA); FB essence (10%; VWR Life Science Seradigm, Radnor, PA, USA); and pen/strep (1%; Corning). For the iMAECs and macrophages utilized within our in vitro experiments, cells were allowed to grow to 70–80% confluency before conducting respective experiments.

### 2.2. iMAEC-Derived Exosome Isolation and Characterization

Exosomes were isolated from serum-free medium conditioned using iMAECs and medium utilized in transwell assays, as described [28]. Briefly, serum-free DMEM was collected, and serial centrifugation was conducted to effectively isolate exosomes from the medium, with the exosomal pellet being resuspended in PBS. Exosome yield and purity was assessed using Coomassie staining (Expedeon, Cambridgeshire, UK), immunoblotting, nanoparticle tracking analyses (NTA) by NanoSight, and transmission electron microscopy (TEM), as previously reported [28].

### 2.3. Generation and Characterization of LP-pXMoAntimiR33a5p

We generated LP-pXMoAntimiR33a5p by adapting the following protocol [21]: Briefly, the following reagents for LP construction were purchased from Avanti Polar Lipids (Alabaster, AL, USA): cholesterol; 1,2-dioleoyl-3-trimethylammonium propane (DOTAP); 1,2-distearoyl-sn-glycero-3-phosphoethanolamine-N-[methoxy(polyethylene glycol)-2000] (DSPE-PEG2k); hydrogenated soy phosphatidylcholine (HSPC). The pUC57-derived plasmid pXMoAntimiR33a5p used to incorporate into LPs was purchased from System Biosciences (Palo Alto, CA, USA) and contains an expression cassette that includes a U6 promoter that drives expression of XMOTIF-tagged anti-miR-33a-5p, along with a TTTTT termination sequence. For each LP-pXMoAntimiR33a5p preparation, 500 mL of pXMoAntimiR33a5p (1 mg/mL) was added to 0.78 mg of DOTAP pre-dissolved in 500 mL of chloroform and 1040 mL of methanol. After gently mixing to form a monophase, we incubated this solution at room temperature for 30 min. Afterward, we added 500 mL of DI water and 500 mL of chloroform to the solution to form an aqueous biphase, where we mixed then spun this solution at 800 g for 10 min at 4 °C. We then added the organic phase to the following dried lipids: 1.47 mg of cholesterol; 0.95 mg of DSPE-PEG2k; 4.55 mg of HSPC. We transferred the mixed organic solution to a sterile tube, added 500 mL of DI water to the sterile tube, vortexed the tube, and then emulsified the solution using sonication. We then used a BUCHI Rotavapor^®^ R-100 (New Castle, DE, USA) for evaporating the organic phase. We subsequently added 500 mL of DI water to the preparation and conducted another round of evaporation using the Rotavapor. We then extruded the LP preparation for a total of 21 passages by using a 100 nm polycarbonate membrane at a temperature of 55 °C. A purified, custom VHPK (Val-His-Pro-Lys-Gln-His-Arg-Gly-Gly-Ser-Lys(stearic_Lys(stearic)-PEG27)-Gly-Cys) purchased from CPC Scientific (San Jose, CA, USA) was used for LP decoration. Ten mg of lyophilized peptide was directly added to the LP preparation and mixed/inverted and stirred with gentle agitation. LP-pXMoAntimiR33a5p was then purified using a Cytiva HiTrap column (Marlborough, MA, USA) and VHPK-rich fractions were assessed using spectrophotometry [34] and HPLC. For HPLC analysis, the chromatographic system used was a ThermoFisher UltiMate 3000 HPLC system (Waltham, MA, USA) with a quaternary pump, a semiautomatic Rheodyne injector with a 20 mL loop, and VWD-3100 detector at 220 nm, and the HPLC column used was a Jupiter C4 300 Å 250 mm × 4.6 mm × 5 um from Phenomenex (Torrance, CA, USA). To assess pXMoAntimiR33a5p incorporation into the LPs, we performed a degradation assay. In brief, LP-pXMoAntimiR33a5p preparations were divided equally into three separate treatment groups and initially incubated for 15 min at room temperature with either PBS (1% total volume) or PBS-T at a 1% total volume to disrupt LPs. The PBS-T treatment group and one of the PBS treatment groups were then treated with DNase I (Promega, Madison, WI, USA), while the other PBS treatment group was incubated with vehicle. After incubating the 3 groups in a water bath at 37 °C for 60 min, DNase I was heat inactivated, and PBS-T was added to each treatment accordingly at a 1% total volume for each group. The plasmid DNA from each treatment was then purified by using a DNA Clean & Concentrator kit (Zymo Research) to use for analyzing pXMoAntimiR33a5p content outside of LPs versus within intact LPs. Additional characterization of the LP-pXMoAntimiR33a5p preparations included assessing the diameter size and physical characteristics of the particles within these preparations using NTA and TEM [35], respectively.

### 2.4. Transwell Assays

To effectively separate basolaterally secreted exosomes from exosomes released apically [36,37] by cultured iMAECs, we utilized a transwell system [36] (0.4 μm pore size; Corning). iMAECs were initially maintained in the apical compartment of the transwell using standard growth medium, and the bottom compartment was filled with PBS. Once iMAECs reached optimal confluency, cells were rinsed with PBS and replenished with standard growth medium containing either vehicle only or lipopolysaccharide (LPS) (10 ng/mL; Sigma-Aldrich, St. Louis, MO, USA). PBS was also removed from the bottom compartment and this compartment was rinsed with PBS, then refilled with PBS. Twenty-four hours after vehicle and LPS treatments, the cells and bottom compartments were rinsed with PBS as described above, the bottom compartment refilled with PBS, and cells exposed to LP-pXMoAntimiR33a5p (1 iMAEC per LP) diluted in standard growth medium. One-hour after incubating iMAECs with LP-pXMoAntimiR33a5p, cells and bottom compartments were again rinsed with PBS as described above, and both compartments were filled with serum-free DMEM. Twenty-four hours later, medium from the apical and basolateral compartments were either used to treat cultured RAW 264.7 macrophages or exosomes from this medium were isolated using ultracentrifugation [28].

### 2.5. qPCR and RT-qPCR

Plasmid internalization efficiency of iMAECs exposed to LP-pXMoAntimiR33a5p was assessed using qPCR. iMAECs were first allowed to grow to optimal confluency, rinsed with PBS, and then replenished with standard growth medium that contained vehicle only or LPS (10 ng/mL). Twenty-four hours after vehicle/LPS treatments, iMAECs were rinsed with PBS, and then incubated with LP-pXMoAntimiR33a5p (1 iMAEC per LP) diluted in standard growth medium. One hour after exposing iMAECs with LP-pXMoAntimiR33a5p, iMAECs were rinsed with PBS and refed with standard growth medium for twenty-four hours, before again rinsing iMAECs with PBS, then extracting gDNA and plasmid with DNA QuickExtract™ DNA Extraction Solution (Middleton, WI, USA). This extracted DNA, along with the purified DNA collected from the LP degradation assays, was used to measure plasmid DNA content by using a primer pair specific for detecting the plasmid, pXMoAntimiR33a5p. To assess miR-33a-5p and anti-miR-33a-5p levels, we used RT-qPCR. Briefly, we purified total RNA from the exosomes isolated from serum-free medium conditioned using iMAECs via ultracentrifugation, as previously described [28]. For cellular RNA extraction, we first treated cultured RAW 264.7 macrophages with serum-free medium conditioned using vehicle/LPS-treated iMAECs exposed to LP-pXMoAntimiR33a5p. Twenty-four hours after this treatment, we rinsed the macrophages with PBS, and purified the total RNA from these cells with a Direct-zol RNA purification kit (Zymo Research, Irvine, CA, USA). We synthesized small RNA using the cellular RNA as template along with a qScript™ microRNA cDNA Synthesis kit (Quantabio, Beverly, MA, USA). A Quantabio PerfeCTa SYBR Green FastMix kit was utilized in all our qPCR reactions [38] and the U6 reference gene was also measured when calculated total RNA input was identical among samples. For experiments involving LP-pXMoAntimiR33a5p degradation assays, measuring pXMoAntimiR33a5p levels in vehicle versus LPS-treated iMAECs incubated with LP-pXMoAntimiR33a5p, and measuring anti-miR-33a-5p content in exosomes secreted basolaterally versus apically in LPS-challenged iMAECs exposed to LP-pXMoAntimiR33a5p, no reference “housekeeping” gene or equal DNA/RNA input was used. Instead, we performed qPCR and RT-qPCR reactions using equal sample volumes and then converted pXMoAntimiR33a5p content and anti-miR-33a-5p expression levels into arbitrary units, as previously shown [28]. We used the following primer pair to detect the plasmid pXMoAntimiR33a5p in our qPCR reactions (fwd: 5′-GCTTAACTATGCGGCATCAGAG-3′; rev: 5′-TAATCGCCTTGCAGCACATC-3′). For small RNA detection, we used the listed forward primers in our qPCR reactions (U6: 5′-TGGCCCCTGCGCAAGGATG-3′; miR-33a-5p: 5′-CGCGTGCATTGTAGTTGCATTGC-3′; anti-miR-33a-5p: 5′-TGCAATGCAACTACAATGCAC-3′). For these primer sets, we used the universal/global small RNA reverse primer in our qPCR reactions (5′-GCATAGACCTGAATGGCGGTA-3′).

### 2.6. SDS-PAGE and Immunoblotting

Lysates derived from cultured cells and exosomes were prepared as described [28,33,35,39] to be used downstream for protein expression analysis. Briefly, iMAECs were treated with either vehicle or LPS (10 ng/mL for 24 h) before rinsing cells with PBS and collecting lysates. RAW 264.7 macrophages were incubated with serum-free medium conditioned using vehicle/LPS-treated iMAECs exposed to LP-pXMoAntimiR33a5p for 48 h, before rinsing macrophages with PBS, and then collecting cell lysates. We also collected lysates from the exosomes isolated from serum-free medium conditioned using iMAECs via ultracentrifugation [28]. Protein quantification of lysates was performed by using a BCA assay kit (BioVision, Milpitas, CA, USA). To assess protein expression patterns in iMAECs and exosomal lysates, proteins were separated on an SDS-PAGE gel followed by Coomassie staining. For immunoblotting, protein lysates were also separated with an SDS-PAGE gel and the proteins were transferred onto a PVDF membrane (Merck Millipore Ltd., Burlington, MA, USA). After incubating PVDF membranes in blocking buffer then washing with TBST, the membranes were incubated with the following primary antibodies: ABCA1 (1:1500 dilution, sc-58219; Santa Cruz Biotechnology, Dallas, TX, USA); calregulin (1:500 dilution, sc-166837, Santa Cruz Biotechnology); CD81 (1:500 dilution, sc-166029; Santa Cruz Biotechnology); VCAM-1 (1:1000 dilution, sc-13160, Santa Cruz Biotechnology); GAPDH (1:2000 dilution, sc-365062; Santa Cruz Biotechnology). Post-incubation with primary antibodies, PVDF membranes were washed with TBST, and then incubated with HRP-conjugated goat anti-mouse IgG secondary antibody (1:15,000 dilution, AP181P; Sigma-Aldrich). After incubating PVDF membranes with secondary antibody and washing with TBST, we used ECL (Immobilon ECL Ultra Western HRP Substrate; MilliporeSigma, Billerica, MA, USA) and a ChemiDoc imager (Analytik Jena US, Upland, CA, USA) for protein detection [38], and then quantified proteins with version 1.53a NIH ImageJ software [40].

### 2.7. ApoAI-Mediated Cholesterol Efflux

RAW 264.7 macrophages were grown in standard growth medium until reaching optimal confluency and then rinsed with PBS and subsequently treated for 24 h with serum-free medium conditioned using vehicle- or LPS-treated iMAECs exposed to LP-pXMoAntimiR33a5p. After treatments, we rinsed the cultured macrophages with PBS, and then loaded the cells with [^3^H] cholesterol (1 μCi/mL; PerkinElmer, Waltham, MA, USA) diluted in serum-free DMEM supplemented with 2 mg/mL of fatty acid-free bovine serum albumin (Sigma-Aldrich). After loading macrophages with [^3^H] cholesterol for 24 h, we rinsed the macrophages with PBS, and incubated the macrophages with 50 μg/mL of apoAI (Academy Bio-Medical Company, Houston, TX, USA) diluted in serum-free DMEM supplemented with fatty acid-free bovine serum albumin (2 mg/mL) for 24 h. We then filtered the medium to remove any dissociated macrophages and counted the [^3^H] in the medium and cell extracts by using a liquid scintillation counter (Tri-Carb 4910TR; PerkinElmer). We calculated apoAI-mediated cholesterol in the treated macrophages as previously described [28,39].

### 2.8. Statistical Analysis

SigmaPlot v14.0 (Systat Software Inc., San Jose, CA, USA) was utilized for statistical analyses. We assessed normality using a Shapiro–Wilk test and assessed equal variance by using a Brown–Forsythe test. For the degradation assay, we performed a Kruskal–Wallis one-way analysis of variance on ranks and Dunn’s method for post-hoc testing. For all other statistical tests, we performed a Student’s *t*-test when both these normality and equal variance assumptions were met, performed a Mann–Whitney rank-sum test when normality was violated, and performed a Welch’s *t*-test when equal variances were not assumed. Statistical significance was set at *p*-value of <0.05.

## 3. Results

### 3.1. iMAECs Secrete Exosomes

**Figure 1:**
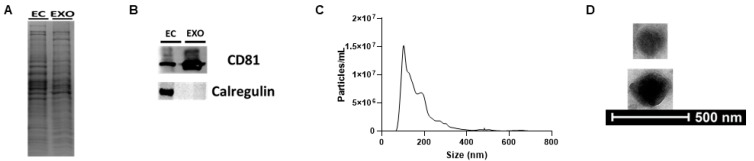
Characterizing iMAEC-derived exosomes. (**A**) SDS-PAGE and Coomassie stain showing distinct protein profiles of iMAEC lysate (EC) and iMAEC-derived exosomal (EXO) lysate. (**B**) Immunoblotting of lysates for iMAECs and iMAEC-derived exosomes for probing of the exosome-positive marker CD81 and exosome-negative marker calregulin. (**C**) Particle number and diameter size determined in iMAEC-derived exosomal preparations via NTA. (**D**) Transmission electron micrographs of exosomes within iMAEC-derived exosomal preparations.

We and others have confirmed that cultured primary aortic endothelial cells secrete exosomes [28,41], but to our knowledge, rigorous analysis for determining whether iMAECs release exosomes has yet to be conducted. Therefore, we performed robust assessments of the particles isolated from the serum-free medium conditioned using iMAECs to confirm whether these isolated particles are exosomes. Coomassie staining of an SDS-PAGE gel revealed differences in protein expression patterns of lysate derived from particles isolated from the conditioned medium of iMAECs when compared to the protein profile of iMAEC lysate (Figure 1A). We used these lysates to probe for the tetraspanin and exosome marker CD81, as well as the endoplasmic reticulum protein and non-exosome marker calregulin. As expected, CD81 protein was enriched within the iMAEC-derived exosomal lysate when compared to iMAEC lysate, while calregulin was detected in iMAEC lysate but absent in iMAEC-derived exosomal lysate (Figure 1B), which is predicted to occur when analyzing exosomal versus cellular lysates [42]. We further characterized these particles secreted by iMAECs using NTA via NanoSight, which showed iMAECs release a large number of particles that are predominantly within the same size range as exosomes [42] (Figure 1C). Lastly, we imaged these particles with TEM, which is considered the gold standard for visualizing exosomes [43], and observed exosome-sized particles with distinctive traits commonly attributed to exosomes [44,45] (Figure 1D). From these findings, we concluded that iMAECs do release exosomes.

### 3.2. Characterization of LP-pXMoAntimiR33a5p

**Figure 2:**
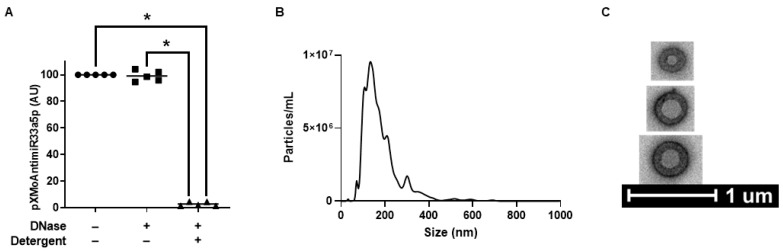
Characterization of LP-pXMoAntimiR33a5p preparations. (**A**) Five LP-pXMoAntimiR33a5p preparations were exposed to either detergent and DNase or DNase only and pXMoAntimiR33a5p content within treated preparations were compared to pXMoAntimiR33a5p levels in vehicle-treated control LP-pXMoAntimiR33a5p via qPCR. AU = arbitrary units. Bars are group means and (*) shows statistical significance at *p* < 0.05. (**B**) Particle diameter size and number assessed in LP-pXMoAntimiR33a5p using NTA. (**C**) TEM images of LP-pXMoAntimiR33a5p.

We performed a degradation assay to assess incorporation of pXMoAntimiR33a5p into LPs. While DNase alone failed to significantly degrade pXMoAntimiR33a5p within LP-pXMoAntimiR33a5p preparations, the addition of detergent to promote LP lysis in LP-pXMoAntimiR33a5p along with DNase resulted in effective pXMoAntimiR33a5p degradation (Figure 2A), which implies pXMoAntimiR33a5p is protected within intact LPs. NTA was utilized to determine size and yield of particles within LP-pXMoAntimiR33a5p preparations, which detected a high number of particles <300 nm in size (Figure 2B). TEM images of the particles within the LP-pXMoAntimiR33a5p preparations revealed particles exhibiting physical characteristics commonly associated with LPs (Figure 2C). Therefore, these results indicate that our preparations contain intact LPs that enclose pXMoAntimiR33a5p.

### 3.3. Proinflammatory iMAECs Exposed to LP-pXMoAntimiR33a5p Primarily Release Anti-miR-33a-5p-Loaded Exosomes Basolaterally

**Figure 3:**
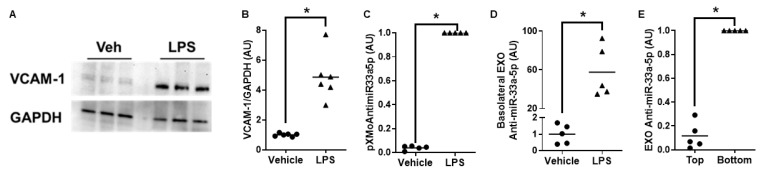
Proinflammatory iMAECs exposed to LP-pXMoAntimiR33a5p package anti-miR-33a-5p in exosomes that are largely released basolaterally. (**A**) Representative immunoblot of VCAM-1 protein for iMAECs treated with vehicle only or challenged with LPS. (**B**) Immunoblot quantification of VCAM-1 protein in iMAECs treated with vehicle only or challenged with LPS with datapoints indicating biological triplicates from two independent experiments. (**C**) Levels of pXMoAntimiR33a5p measured via qPCR in iMAECs pretreated with vehicle only or challenged with LPS and then incubated with LP-pXMoAntimiR33a5p. (**D**) Anti-miR-33a-5p content in exosomes (EXO) secreted basolaterally by iMAECs pretreated with vehicle only or challenged with LPS and then exposed to LP-pXMoAntimiR33a5p. (**E**) Anti-miR-33a-5p levels assessed in EXO secreted apically (top) or basolaterally (bottom) using LPS-challenged iMAECs treated with LP-pXMoAntimiR33a5p. (**D**,**E**) Anti-miR-33a-5p measured via RT-qPCR. (**C**–**E**) Each data point indicates one independent experiment. (**B**–**E**) AU = arbitrary units. Bars are group means and (*) shows statistical significance at *p* < 0.05.

To induce VCAM-1 expression in iMAECs, we challenged cells with LPS [46]. Compared to vehicle-treated control iMAECs, LPS-challenged iMAECs showed robust expression of VCAM-1 protein (Figure 3A,B). When we exposed vehicle-treated and LPS-challenged iMAECs to LP-pXMoAntimiR33a5p, we observed a significant increase in pXMoAntimiR33a5p content within iMAECs challenged with LPS (Figure 3C), which implies LP-pXMoAntimiR33a5p is selectively internalized by proinflammatory endothelial cells. To determine whether this increase in pXMoAntimiR33a5p content also resulted in increasing anti-miR-33a-5p levels in exosomes released basolaterally by LPS-challenged iMAECs, we measured basolateral exosomal anti-miR-33a-5p levels in LPS-challenged iMAECs versus vehicle control iMAECs. We reported a significant increase in basolateral exosomal anti-miR-33a-5p content in the LPS-challenged iMAEC group when compared to the vehicle control iMAEC group (Figure 3D). And to assess whether proinflammatory iMAECs largely secrete exosomes apically or basolaterally, we measured anti-miR-33a-5p levels in exosomes released basolaterally versus apically from LPS-challenged iMAECs incubated with LP-pXMoAntimiR33a5p. In this experiment, we observed a significant increase in anti-miR-33a-5p content from the exosomes secreted basolaterally compared to the exosomes released apically (Figure 3E). Taken together, these results demonstrate that proinflammatory iMAECs exposed to LP-pXMoAntimiR33a5p package anti-miR-33a-5p in exosomes that are preferentially secreted basolaterally.

### 3.4. ABCA1-Dependent Cholesterol Efflux Is Enhanced in Macrophages Exposed to Exosomes Secreted Basolaterally by Proinflammatory Endothelial Cells Incubated with LP-pXMoAntimiR33a5p

**Figure 4:**
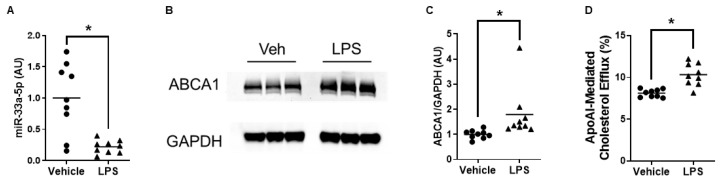
Increased ABCA1-dependent cholesterol efflux occurs in macrophages that internalize anti-miR-33a-5p-filled exosomes secreted basolaterally by proinflammatory iMAECs incubated with LP-pXMoAntimiR33a5p. (**A**–**D**) Raw 264.7 macrophages were treated with serum-free medium containing exosomes released basolaterally by iMAECs pretreated with either vehicle only or challenged with LPS and then subsequently exposed to LP-pXMoAntimiR33a5p. (**A**) MiR-33a-5p expression measured in treated macrophages via RT-qPCR. (**B**) Representative immunoblot of ABCA1 protein for treated macrophages. (**C**) Immunoblot quantification of ABCA1 protein in treated macrophages. (**D**) ApoAI-mediated cholesterol efflux measured in treated macrophages. (**A**,**C**) AU = arbitrary units. (**A**,**C**,**D**) Datapoints indicate biological triplicates from three independent experiments. Bars are group means and (*) shows statistical significance at *p* < 0.05.

We incubated cultured macrophages with medium containing exosomes secreted basolaterally by vehicle/LPS-treated iMAECs exposed to LP-pXMoAntimiR33a5p to assess the impact of miR-33a-5p expression within these treated macrophages. The macrophages exposed to basolateral medium associated with LPS-challenged iMAECs showed significant decreases in miR-33a-5p expression when compared to cultured macrophages incubated with the basolateral medium associated with vehicle-treated iMAECs (Figure 4A). We also observed a significant increase in ABCA1 protein expression in the macrophages incubated with basolateral medium associated with LPS-challenged iMAECs when compared to macrophages exposed to basolateral medium associated with vehicle-treated iMAECs (Figure 4B,C). The macrophages incubated with basolateral medium associated with LPS-challenged iMAECs also exhibited enhanced apoAI-mediated cholesterol efflux versus cultured macrophages exposed to basolateral medium associated with vehicle-treated iMAECs (Figure 4D). Therefore, these results suggest that enhanced ABCA1-dependent cholesterol efflux occurs in macrophages that internalize anti-miR-33a-5p-filled exosomes released basolaterally by inflamed endothelia that incorporated LP-pXMoAntimiR33a5p.

## 4. Discussion

**Figure 5:**
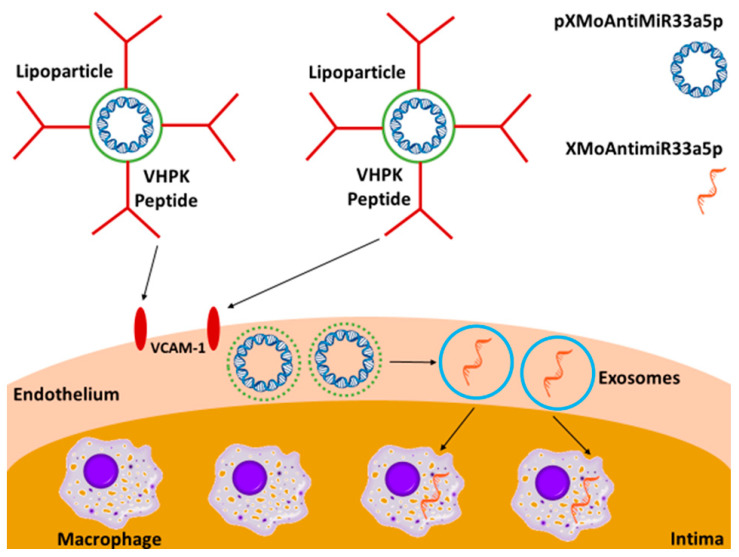
Working model illustrating proinflammatory endothelial cell-derived exosome-mediated delivery of anti-miR-33a-5p to lipid-laden arterial intimal macrophages instigated using VCAM-1-binding lipoparticles containing pXMoAntimiR33a5p.

In this study, we wanted to investigate whether treating proinflammatory iMAECs with LP-pXMoAntimiR33a5p results in these cells releasing anti-miR-33a-5p-loaded exosomes basolaterally. However, a prerequisite for this is to determine if iMAECs are capable of secreting exosomes, since immortalized cell lines are known to behave differently when compared to their primary cell counterparts [47]. Therefore, we performed some initial sets of experiments to confirm that iMAECs do release exosomes. We also showed when iMAECs are exposed to LP-pXMoAntimiR33a5p; these VCAM-1-binding LPs selectively target proinflammatory iMAECs, and the iMAECs that internalize LP-pXMoAntimiR33a5p preferentially secrete anti-miR-33a-5p-filled exosomes basolaterally. Lastly, when we incubated cultured macrophages with medium containing anti-miR-33a-5p-loaded exosomes released basolaterally by proinflammatory iMAECs exposed to LP-pXMoAntimiR33a5p, the treated macrophages exhibited increased ABCA1-dependent cholesterol efflux. Thus, these proof-of-concept experiments imply anti-miR-33a-5p delivery to intimal macrophages is possible by combining lipoparticle- and exosome-based approaches (Figure 5).

Cardiovascular disease is still the leading cause of global mortality [48] and atherosclerosis is the major contributor for these deaths [49]. Unfortunately, treatments that have focused on attenuating the main two drivers of atherosclerosis, vascular inflammation and arterial cholesterol accumulation, remain suboptimal, as they only appear to demonstrate partial efficacy [50,51,52,53]. More effective therapies geared toward cholesterol removal from arterial lesions would likely require the promotion of cholesterol efflux from intimal lipid-laden macrophages, as these cells are considered the main culprit in the development of atherosclerosis [54]. However, precisely targeting these cells remains challenging due to these lipid-laden macrophages being located within the intimal of atherosclerotic lesions with the endothelium acting as a barrier [55], which prevents therapeutic entry into the intima.

Nanotherapy is an attractive option for atherosclerosis [56,57] because it allows transgenes and other therapeutic bioactives to be precisely delivered to atherosclerotic lesions. For instance, nanoparticles that target macrophages have shown efficacy in treating atherosclerosis in preclinical animal models [58,59]. However, this type of therapeutic intervention would likely only be able to treat advanced atherosclerotic lesions that trigger the development of leaky endothelium [60,61,62,63], because nanoparticles are considered too large to bypass intact endothelia and reach lipid-laden intimal macrophages.

Since the intima lies directly underneath the endothelial cells, strategies that focus on proinflammatory endothelia to deliver transgenes to intimal cells is a promising atheroprotective approach. One potential way to accomplish this is by delivering XMOTIF-tagged transgenes to endothelial cells, as we and others have shown XMOTIF-tagged small RNA transgenes that are introduced to endothelial cells and other cell types results in these transgenes being packaged into exosomes [26,27,28]. In our study, we constructed VCAM-1-binding LP-pXMoAntimiR33a5p to precisely target proinflammatory endothelial cells so that XMOTIF-tagged anti-miR-33a-5p could be shuttled into endothelial cell-derived exosomes; thus, these exosomes could be exploited as a form of atheroprotection. However, this strategy could be expanded to include different plasmids that express other XMOTIF-tagged transgenes, as well as XMOTIF-tagged siRNA/shRNA. Indeed, by incorporating this transgenic material into different types of cell-targeting nanoparticles, there is potential for effectively delivering small RNA transgenes to various cells through exosome-mediated processes, which opens the possibility for potentially treating other diseases besides atherosclerosis.

Herein, we want to highlight the limitations of this study. One potential limitation is using immortalized cells instead of primary cells in our sets of experiments, as immortalized cells may behave unlike primary cells and respond to various interventions differently [47]. The macrophage cell line we selected in this study was RAW 264.7 [64], and in a prior study involving endothelial cell-derived exosome-mediated transfer of anti-miR-33a-5p to macrophages, THP-1 cells were used [28]. Future studies should be conducted to determine whether exosome-mediated delivery of anti-miR-33a-5p to primary macrophages also results in enhancing ABCA1-dependent cholesterol efflux. In addition, iMAECs may also react differently to LP-pXMoAntimiR33a5p and proinflammatory stimuli when compared to primary arterial endothelial cells. For instance, while we observed a striking increase in basolateral exosomal anti-miR-33a-5p levels when compared to apical anti-miR-33a-5p levels when anti-miR-33a-5p content was assessed in LPS-challenged iMAECs incubated with LP-pXMoAntimiR33a5p, it is possible that a more bidirectional pattern of exosome release may occur if primary arterial endothelial cells are exposed to these same conditions. Thus, future studies should be devoted to directly testing whether exosomes from primary arterial proinflammatory endothelial cells predominantly secrete exosomes basolaterally, so that these exosomes may be exploited as atheroprotective agents in vivo. Lastly, it may be possible that the increase in ABCA1 protein expression observed in macrophages from anti-miR-33a-5p inhibition may have atheroprotective properties that extend beyond enhancing apoAI-mediated cholesterol efflux. For instance, ABCA1 has been shown to have the capacity to participate in HDL-mediated cholesterol efflux and demonstrate anti-inflammatory effects [39,65,66,67]. Future experiments should analyze if endothelial cell-derived exosome-mediated transfer of anti-miR-33a-5p to macrophages may also exhibit any other atheroprotective qualities outside of apoAI-mediated cholesterol efflux. Lastly, two other limitations in our study are not extensively characterizing the lipoparticles [21] and not directly analyzing LP-pXMoAntimiR33a5p stability. While our current data indirectly suggest that LP-pXMoAntimiR33a5p are stable in cultured conditions, these particles will need to be resistant to degradation when administered to animal models and remain stable in vivo to demonstrate any beneficial atheroprotective effect. With this in mind, future studies involving atherogenic animal models should also assess LP-pXMoAntimiR33a5p stability.

As we envision transitioning into testing our LP-pXMoAntimiR33a5p delivery method in vivo for atheroprotective efficacy, there are a few vital factors to point out. Performing partial carotid ligation surgery in mice causes mouse carotid arteries to become highly inflamed, which results in robust VCAM-1 expression and also promotes the rapid development of atherosclerotic lesions within the carotid arteries [21]. Using this strategy would be optimal to test whether administering (e.g., tail vein injection) LP-pXMoAntimiR33a5p to experimental mice post-ligation results in promoting atherosclerosis regression within their carotid arteries. Another approach would be to test whether injecting LP-pXMoAntimiR33a5p into mice immediately before and subsequently after partial carotid ligation surgery may prevent carotid artery atherogenesis. From a clinical perspective, dosage frequency should also be taken into consideration when testing efficacy of LP-pXMoAntimiR33a5p in atherogenic animal models. Since LPs will most likely need to be injected intravenously, a weekly injection plan would be ideal, though 2–3 times a week may offer more atheroprotective benefit. Hence, various injection regimens should be initially tested in atherogenic mice to determine which protocol provides the most atheroprotection. If administering LP-pXMoAntimiR33a5p in vivo does result in an antiatherogenic effect, then experiments directly testing whether atheroprotection occurs from exosome-mediated transfer of anti-miR-33a-5p to intimal macrophages should be conducted. This can be accomplished by coupling laser capture microdissection with (RT-)qPCR [68] so that collection of arterial endothelium and intimal macrophages can be performed, followed by DNA/RNA isolation. This extracted nucleic acid can be subsequently used for PCR reactions to test whether pXMoAntimiR33a5p is exclusively found within arterial endothelium, while anti-miR-33a-5p detection primarily occurs in intimal macrophages. Furthermore, if miR-33a-5p expression is shown to be reduced within the intimal macrophages derived from mice injected with LP-pXMoAntimiR33a5p that have been shown to be protected from developing atherosclerosis, then this would offer compelling evidence that the atheroprotective effect observed in these mice is mediated by miR-33a-5p inhibition within these cells. Safety of LP-pXMoAntimiR33a5p is a concern as well and if administering LP-pXMoAntimiR33a5p to atherogenic animal models is atheroprotective, then rigorously testing whether injecting these particles in vivo is safe should be performed by conducting safety profile experiments in addition to performing LP-pXMoAntimiR33a5p biodistribution and clearance experiments as previously described [21].

In conclusion, LP-pXMoAntimiR33a5p appears to be a promising agent for treating atherosclerosis, as this therapeutic has the potential to facilitate exosome-mediated transfer of anti-miR-33a-5p to lipid-laden intimal cells when internalized by proinflammatory endothelial cells. Future directions should focus on in vivo studies that directly test whether treating atherogenic animal models with LP-pXMoAntimiR33a5p results in atheroprotection.

## Data Availability

All the data represented in this study are provided within the manuscript.

## References

[1] Pahwa R., Jialal I. (2020). StatPearls (Atherosclerosis). StatPearls.

[2] Lusis A.J. (2000). Atherosclerosis. Nature.

[3] Naghavi M., Libby P., Falk E., Casscells S.W., Litovsky S., Rumberger J., Badimon J.J., Stefanadis C., Moreno P., Pasterkamp G. (2003). From vulnerable plaque to vulnerable patient: A call for new definitions and risk assessment strategies: Part I. Circulation.

[4] Hansson G.K., Libby P., Tabas I. (2015). Inflammation and plaque vulnerability. J. Intern. Med..

[5] Wu M.-Y., Li C.-J., Hou M.-F., Chu P.-Y. (2017). New Insights into the Role of Inflammation in the Pathogenesis of Atherosclerosis. Int. J. Mol. Sci..

[6] Ohashi R., Mu H., Wang X., Yao Q., Chen C. (2005). Reverse cholesterol transport and cholesterol efflux in atherosclerosis. Qjm Int. J. Med..

[7] Nakayama A., Albarrán-Juárez J., Liang G., Roquid K.A., Iring A., Tonack S., Chen M., Müller O.J., Weinstein L.S., Offermanns S. (2020). Disturbed flow–induced Gs-mediated signaling protects against endothelial inflammation and atherosclerosis. J. Clin. Investig..

[8] Chiu J.-J., Chien S., Venturini G., Malagrino P.A., Padilha K., Tanaka L.Y., Laurindo F.R., Dariolli R., Carvalho V.M., Cardozo K.H.M. (2011). Effects of Disturbed Flow on Vascular Endothelium: Pathophysiological Basis and Clinical Perspectives. Physiol. Rev..

[9] Liao J.K. (2013). Linking endothelial dysfunction with endothelial cell activation. J. Clin. Investig..

[10] Boulanger C.M. (2018). Highlight on Endothelial Activation and Beyond. Arter. Thromb. Vasc. Biol..

[11] Davignon J., Ganz P. (2004). Role of endothelial dysfunction in atherosclerosis. Circulation.

[12] Gimbrone M.A., Garcia-Cardena G. (2016). Endothelial Cell Dysfunction and the Pathobiology of Atherosclerosis. Circ. Res..

[13] Cook-Mills J.M., Marchese M.E., Abdala-Valencia H. (2011). Vascular Cell Adhesion Molecule-1 Expression and Signaling During Disease: Regulation by Reactive Oxygen Species and Antioxidants. Antioxid. Redox Signal..

[14] Meerschaert J., Furie M.B. (1995). The adhesion molecules used by monocytes for migration across endothelium include CD11a/CD18, CD11b/CD18, and VLA-4 on monocytes and ICAM-1, VCAM-1, and other ligands on endothelium. J. Immunol..

[15] Gerhardt T., Ley K. (2015). Monocyte trafficking across the vessel wall. Cardiovasc. Res..

[16] Libby P., Ridker P.M., Maseri A. (2002). Inflammation and atherosclerosis. Circulation.

[17] Libby P. (2012). Inflammation in atherosclerosis. Arterioscler. Thromb. Vasc. Biol..

[18] Libby P. (2002). Inflammation in atherosclerosis. Nature.

[19] Thayse K., Kindt N., Laurent S., Carlier S. (2020). VCAM-1 Target in Non-Invasive Imaging for the Detection of Atherosclerotic Plaques. Biology.

[20] Khodabandehlou K., Masehi-Lano J.J., Poon C., Wang J., Chung E.J. (2017). Targeting cell adhesion molecules with nanoparticles using in vivo and flow-based in vitro models of atherosclerosis. Exp. Biol. Med..

[21] Kheirolomoom A., Kim C.W., Seo J.W., Kumar S., Son D.J., Gagnon M.K., Ingham E.S., Ferrara K.W., Jo H. (2015). Multifunctional Nanoparticles Facilitate Molecular Targeting and miRNA Delivery to Inhibit Atherosclerosis in ApoE^−/−^ Mice. ACS Nano.

[22] Dosta P., Tamargo I., Ramos V., Kumar S., Kang D.W., Borros S., Jo H. (2021). Delivery of Anti-microRNA-712 to Inflamed Endothelial Cells Using Poly(beta-amino ester) Nanoparticles Conjugated with VCAM-1 Targeting Peptide. Adv. Healthc. Mater..

[23] Moore K.J., Tabas I. (2011). The Cellular Biology of Macrophages in Atherosclerosis. Cell.

[24] Bi L., Wacker B.K., Stamatikos A., Sethuraman M., Komandur K., Dichek D.A. (2021). Jugular Vein Injection of High-Titer Lentiviral Vectors Does Not Transduce the Aorta—Brief Report. Arter. Thromb. Vasc. Biol..

[25] Jiang B., Qian K., Du L., Luttrell I., Chitaley K., Dichek D.A. (2011). Helper-Dependent Adenovirus is Superior to First-Generation Adenovirus for Expressing Transgenes in Atherosclerosis-Prone Arteries. Arter. Thromb. Vasc. Biol..

[26] Peterson M.F., Otoc N., Sethi J.K., Gupta A., Antes T.J. (2015). Integrated systems for exosome investigation. Methods.

[27] Fujii N., Hirata H., Ueno K., Mori J., Oka S., Shimizu K., Kawai Y., Inoue R., Yamamoto Y., Matsumoto H. (2017). Extracellular miR-224 as a prognostic marker for clear cell renal cell carcinoma. Oncotarget.

[28] Stamatikos A., Knight E., Vojtech L., Bi L., Wacker B.K., Tang C., Dichek D.A. (2020). Exosome-Mediated Transfer of Anti-miR-33a-5p from Transduced Endothelial Cells Enhances Macrophage and Vascular Smooth Muscle Cell Cholesterol Efflux. Hum. Gene Ther..

[29] Najafi-Shoushtari S.H., Kristo F., Li Y., Shioda T., Cohen D.E., Gerszten R.E., Näär A.M. (2010). MicroRNA-33 and the SREBP Host Genes Cooperate to Control Cholesterol Homeostasis. Science.

[30] Rayner K.J., Suárez Y., Dávalos A., Parathath S., Fitzgerald M.L., Tamehiro N., Fisher E.A., Moore K.J., Fernández-Hernando C. (2010). MiR-33 Contributes to the Regulation of Cholesterol Homeostasis. Science.

[31] Rayner K.J., Sheedy F.J., Esau C.C., Hussain F.N., Temel R.E., Parathath S., van Gils J.M., Rayner A.J., Chang A.N., Suarez Y. (2011). Antagonism of miR-33 in mice promotes reverse cholesterol transport and regression of atherosclerosis. J. Clin. Investig..

[32] Ni C.-W., Kumar S., Ankeny C.J., Jo H. (2014). Development of immortalized mouse aortic endothelial cell lines. Vasc. Cell.

[33] Huang K., Jo H., Echesabal-Chen J., Stamatikos A. (2021). Combined LXR and RXR Agonist Therapy Increases ABCA1 Protein Expression and Enhances ApoAI-Mediated Cholesterol Efflux in Cultured Endothelial Cells. Metabolites.

[34] Bazban-Shotorbani S., Khare H.A., Kajtez J., Basak S., Lee J.H., Kamaly N. (2021). Effect of Nanoparticle Biophysicochemical Properties on Binding and Transport across Cardiovascular Endothelial Dysfunction Models. ACS Appl. Nano Mater..

[35] Huang K., Garimella S., Clay-Gilmour A., Vojtech L., Armstrong B., Bessonny M., Stamatikos A. (2022). Comparison of Human Urinary Exosomes Isolated via Ultracentrifugation Alone versus Ultracentrifugation Followed by SEC Column-Purification. J. Pers. Med..

[36] Wang X., Wilkinson R., Kildey K., Ungerer J.P.J., Hill M.M., Shah A.K., Mohamed A., Dutt M., Molendijk J., Healy H. (2021). Molecular and functional profiling of apical versus basolateral small extracellular vesicles derived from primary human proximal tubular epithelial cells under inflammatory conditions. J. Extracell. Vesicles.

[37] Thayanithy V., O’hare P., Wong P., Zhao X., Steer C.J., Subramanian S., Lou E. (2017). A transwell assay that excludes exosomes for assessment of tunneling nanotube-mediated intercellular communication. Cell Commun. Signal..

[38] Esobi I.C., Barksdale C., Heard-Tate C., Powell R.R., Bruce T.F., Stamatikos A. (2021). MOVAS Cells: A Versatile Cell Line for Studying Vascular Smooth Muscle Cell Cholesterol Metabolism. Lipids.

[39] Stamatikos A., Dronadula N., Ng P., Palmer D., Knight E., Wacker B.K., Tang C., Kim F., Dichek D.A. (2019). ABCA1 Overexpression in Endothelial Cells In Vitro Enhances ApoAI-Mediated Cholesterol Efflux and Decreases Inflammation. Hum. Gene Ther..

[40] Schneider C.A., Rasband W.S., Eliceiri K.W. (2012). NIH Image to ImageJ: 25 Years of image analysis. Nat. Methods.

[41] Fujimoto S., Fujita Y., Kadota T., Araya J., Kuwano K. (2021). Intercellular Communication by Vascular Endothelial Cell-Derived Extracellular Vesicles and Their MicroRNAs in Respiratory Diseases. Front. Mol. Biosci..

[42] Conde-Vancells J., Rodriguez-Suarez E., Embade N., Gil D., Matthiesen R., Valle M., Elortza F., Lu S.C., Mato J.M., Falcon-Perez J.M. (2008). Characterization and Comprehensive Proteome Profiling of Exosomes Secreted by Hepatocytes. J. Proteome Res..

[43] Kotrbová A., Štěpka K., Maška M., Pálenik J.J., Ilkovics L., Klemová D., Kravec M., Hubatka F., Dave Z., Hampl A. (2019). TEM ExosomeAnalyzer: A computer-assisted software tool for quantitative evaluation of extracellular vesicles in transmission electron microscopy images. J. Extracell. Vesicles.

[44] Nilsson J., Skog J., Nordstrand A., Baranov V., Mincheva-Nilsson L., Breakefield X.O., Widmark A. (2009). Prostate cancer-derived urine exosomes: A novel approach to biomarkers for prostate cancer. Br. J. Cancer.

[45] Vojtech L., Woo S., Hughes S., Levy C., Ballweber L., Sauteraud R.P., Strobl J., Westerberg K., Gottardo R., Tewari M. (2014). Exosomes in human semen carry a distinctive repertoire of small non-coding RNAs with potential regulatory functions. Nucleic Acids Res..

[46] Wong D., Dorovini-Zis K. (1995). Expression of Vascular Cell Adhesion Molecule-1 (VCAM-1) by Human Brain Microvessel Endothelial Cells in Primary Culture. Microvasc. Res..

[47] Kaur G., Dufour J.M. (2012). Cell lines: Valuable tools or useless artifacts. Spermatogenesis.

[48] Roth G.A., Mensah G.A., Johnson C.O., Addolorato G., Ammirati E., Baddour L.M., Barengo N.C., Beaton A.Z., Benjamin E.J., Benziger C.P. (2020). Global Burden of Cardiovascular Diseases and Risk Factors, 1990–2019: Update from the GBD 2019 Study. J. Am. Coll. Cardiol..

[49] Barquera S., Pedroza-Tobías A., Medina C., Hernández-Barrera L., Bibbins-Domingo K., Lozano R., Moran A.E. (2015). Global Overview of the Epidemiology of Atherosclerotic Cardiovascular Disease. Arch. Med Res..

[50] Waters D.D., Hsue P.Y. (2017). PCSK9 Inhibition to Reduce Cardiovascular Risk: Tempering Expectations. Circ. Res..

[51] Thompson P.L., Nidorf S.M. (2018). Anti-inflammatory therapy with canakinumab for atherosclerotic disease: Lessons from the CANTOS trial. J. Thorac. Dis..

[52] Sabatine M.S., Giugliano R.P., Keech A.C., Honarpour N., Wiviott S.D., Murphy S.A., Kuder J.F., Wang H., Liu T., Wasserman S.M. (2017). Evolocumab and Clinical Outcomes in Patients with Cardiovascular Disease. N. Engl. J. Med..

[53] Ridker P.M., Everett B.M., Thuren T., MacFadyen J.G., Chang W.H., Ballantyne C., Fonseca F., Nicolau J., Koenig W., Anker S.D. (2017). Antiinflammatory Therapy with Canakinumab for Atherosclerotic Disease. N. Engl. J. Med..

[54] Tabas I., Bornfeldt K.E. (2016). Macrophage Phenotype and Function in Different Stages of Atherosclerosis. Circ. Res..

[55] Mundi S., Massaro M., Scoditti E., Carluccio M.A., van Hinsbergh V.W.M., Iruela-Arispe M.L., De Caterina R. (2018). Endothelial permeability, LDL deposition, and cardiovascular risk factors-a review. Cardiovasc. Res..

[56] Hossaini Nasr S., Huang X. (2021). Nanotechnology for Targeted Therapy of Atherosclerosis. Front. Pharmacol..

[57] Darwitan A., Wong Y.S., Nguyen L.T.H., Czarny B., Vincent A., Nedumaran A.M., Tan Y.F., Muktabar A., Tang J.K., Ng K.W. (2020). Liposomal Nanotherapy for Treatment of Atherosclerosis. Adv. Health Mater..

[58] Nie S., Zhang J., Martinez-Zaguilán R., Sennoune S., Hossen M.N., Lichtenstein A.H., Cao J., Meyerrose G.E., Paone R., Soontrapa S. (2015). Detection of atherosclerotic lesions and intimal macrophages using CD36-targeted nanovesicles. J. Control. Release.

[59] Zhang J., Nie S., Zu Y., Abbasi M., Cao J., Li C., Wu D., Labib S., Brackee G., Shen C.-L. (2019). Anti-atherogenic effects of CD36-targeted epigallocatechin gallate-loaded nanoparticles. J. Control. Release.

[60] Rodrigues S.F., Granger D.N. (2015). Blood cells and endothelial barrier function. Tissue Barriers.

[61] Peng Z., Shu B., Zhang Y., Wang M. (2019). Endothelial Response to Pathophysiological Stress. Arter. Thromb. Vasc. Biol..

[62] Claesson-Welsh L., Dejana E., McDonald D.M. (2021). Permeability of the Endothelial Barrier: Identifying and Reconciling Controversies. Trends Mol. Med..

[63] Baumer Y., McCurdy S., Weatherby T.M., Mehta N.N., Halbherr S., Halbherr P., Yamazaki N., Boisvert W.A. (2017). Hyperlipidemia-induced cholesterol crystal production by endothelial cells promotes atherogenesis. Nat. Commun..

[64] Lai L., Azzam K.M., Lin W.-C., Rai P., Lowe J.M., Gabor K.A., Madenspacher J.H., Aloor J.J., Parks J.S., Näär A.M. (2016). MicroRNA-33 Regulates the Innate Immune Response via ATP Binding Cassette Transporter-mediated Remodeling of Membrane Microdomains. J. Biol. Chem..

[65] Esobi I., Olanrewaju O., Echesabal-Chen J., Stamatikos A. (2022). Utilizing the LoxP-Stop-LoxP System to Control Transgenic ABC-Transporter Expression In Vitro. Biomolecules.

[66] Heinecke J.W. (2015). Small HDL promotes cholesterol efflux by the ABCA1 pathway in macrophages: Implications for therapies targeted to HDL. Circ. Res..

[67] Du X.-M., Kim M.-J., Hou L., Le Goff W., Chapman M.J., Van Eck M., Curtiss L.K., Burnett J.R., Cartland S.P., Quinn C.M. (2015). HDL Particle Size Is a Critical Determinant of ABCA1-Mediated Macrophage Cellular Cholesterol Export. Circ. Res..

[68] Feig J.E., Fisher E.A. (2013). Laser Capture Microdissection for Analysis of Macrophage Gene Expression from Atherosclerotic Lesions. Laser Capt. Microdissect. Methods Protoc..

